# Supplementary material for: The Dilated Cardiomyopathy-Causing Mutation ACTC E361G in Cardiac Muscle Myofibrils Specifically Abolishes Modulation of Ca2+ Regulation by Phosphorylation of Troponin I
Source: Biophys J. 2014 Nov 18;107(10):2369–80. doi: 10.1016/j.bpj.2014.10.024 (PMC4241448; doi:10.1016/j.bpj.2014.10.024)
Supplement: Document S1. Five figures [file mmc1.pdf]

SUPPORTING MATERIAL

2014BIOPHYSJ304024:

"The DCM-causing mutation ACTC E361G in cardiac muscle myofibrils specifically abolishes modulation of Ca2+-regulation by phosphorylation of troponin I"

Petr G. Vikhorev, Weihua Song, Ross Wilkinson, O'Neal Copeland, Andrew E. Messer, Michael A. Ferenczi, Steven B. Marston

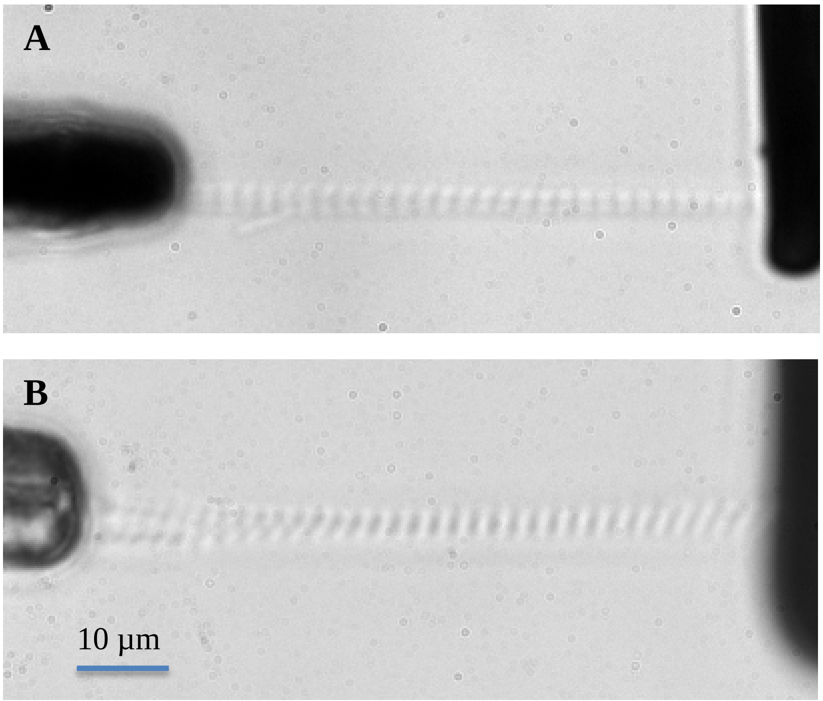

FIGURE S1

A small bundle of myofibrils positioned horizontally between two microneedles

A small group of myofibrils (3-4 myofibrils; *A*, wild-type; *B*, *ACTC* E361G) arranged in a stack were pre-stretched to a SL of 2.17 µm. Due to force probe compliance, the average SL of contracted myofibrils at maximum force became 2.07 and 1.82 µm, respectively for the myofibrils pre-stretch to a SL of 2.17 and 1.90. The changes in SL were about 2-6% in all conditions.

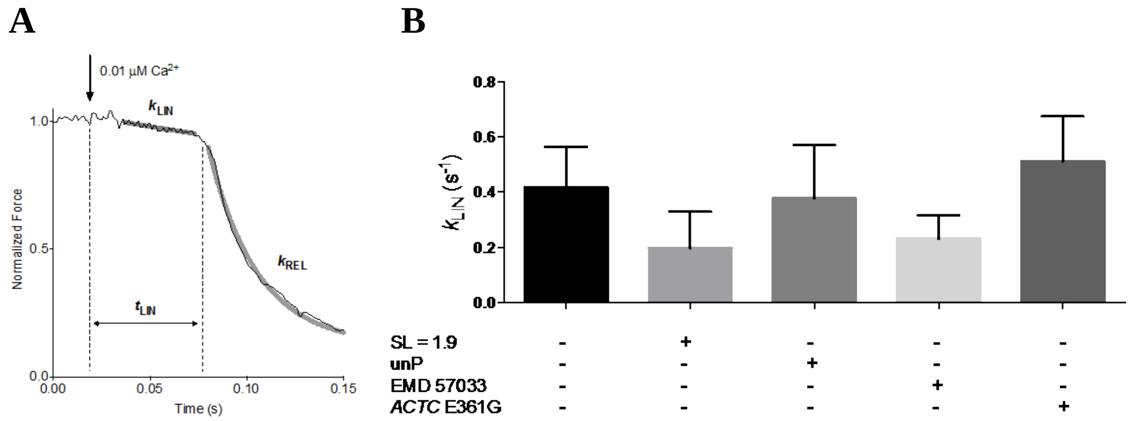

FIGURE S2

The effect of SL change, dephosphorylation, EMD 57033 and mutation *ACTC* E361G on the parameter k_{LIN}.

(*A*) During the slow relaxation phase an active tension is essentially not changing. After a certain time lag (t_{LIN}) the slow phase (characterized also by a rate constant k_{LIN}) is followed by a rapid exponential force decay with a rate constant k_{REL}. A short transition period can be seen during which the system shifts from the linear phase to the exponential. The transition phase was not entered neither the regression line calculation nor the exponential. The k_{LIN} was calculated from the slope of the linear fit (k_{LIN}=slope/Fmax).

(*B*) The k_{LIN} measured in different conditions, compared to control (first black column in *B*; NTG myofibrils, SL = 2.17, phosphorylated, untreated). Values are means ± SE, n = 5-9. There was no difference ($p \geq 0.35$) in k_{LIN}.

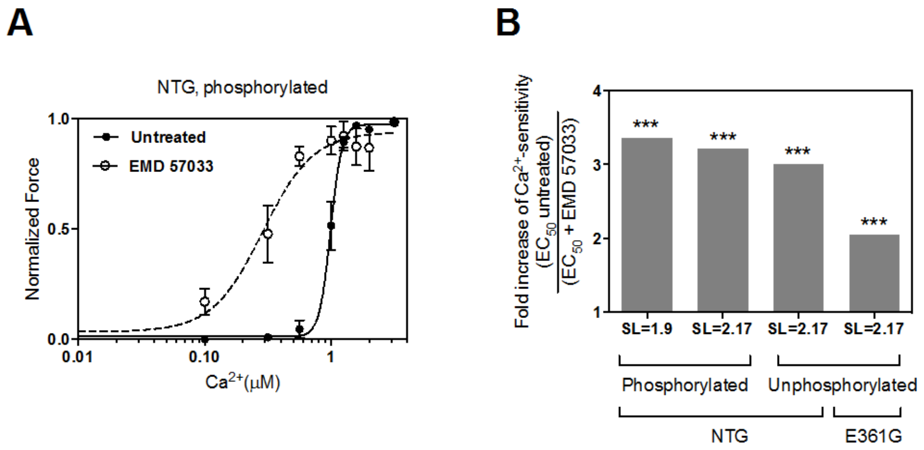

FIGURE S3

The effect of EMD57033 on mouse myofibril contractility

(*A*) The effect of EMD57033 on force-Ca^{2+} relationship. The Ca^{2+}-force sensitivity curve for treated myofibrils (*open circles*, *dashed line*) is shifted to the left, as compared to untreated NTG myofibrils (*solid circles*, *solid line*). Sarcomere length was 2.17 µm. (*B*) The increase in Ca^{2+} sensitivity ($1/EC_{50}$) resulting from incubation with EMD57033. Values are means. ***P < 0.001. Data from Table 1.

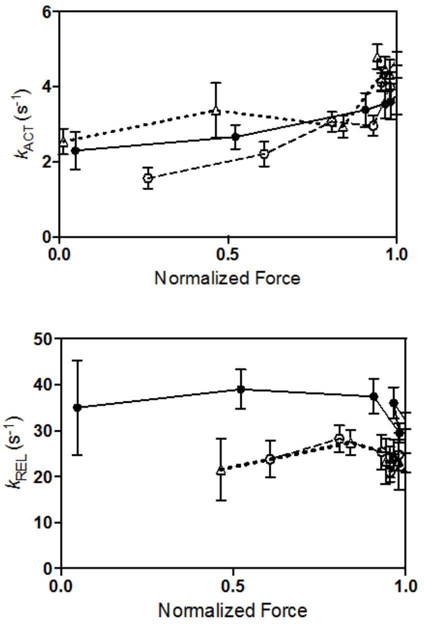

FIGURE S4

Relationship of myofibril contraction and relaxation rates to developed force

Relationship between Force and k_{ACT} and k_{REL} for phosphorylated NTG myofibrils (*solid circles*, *solid line*), unphosphorylated NTG myofibrils (*open circles*, *dashed line*) and phosphorylated *ACTC* E361G myofibrils (*open triangles*, *dotted line*) at SL=2.17. Values are means ± SE.

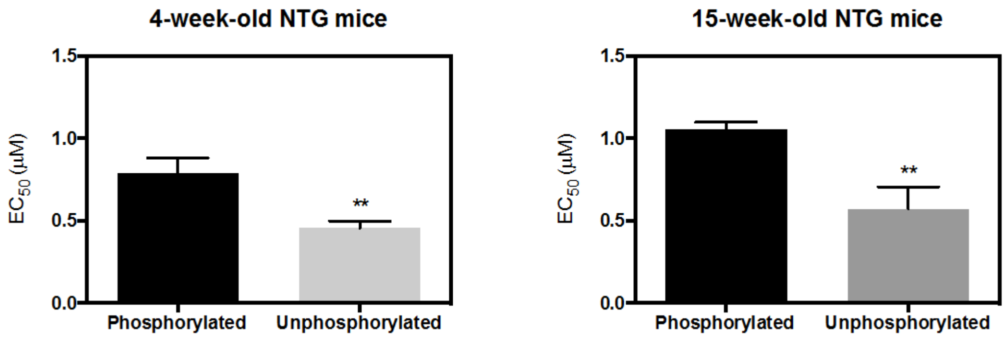

FIGURE S5

The effect of mouse age on myofibril Ca^{2+}-sensitivity

Comparison of EC_{50} values between phosphorylated and unphosphorylated myofibrils from 4-week-old (*A*) and 28-week-old (*B*). Values are means ± SE, n = 5-8. **P < 0.01.

Phosphorylated myofibrils from 4-week-old mouse had slightly higher calcium sensitivity of force than myofibrils obtained from 28-week-old mouse (0.79 ± 0.09 vs. 1.05 ± 0.04 µM, p = 0.02). There was no difference in *n*H. Dephosphorylation of sarcomere proteins increased calcium sensitivity by 1.73 and 1.84 fold in 4-week-old and 28-week-old mice, respectively. The age difference between unphosphorylated samples was not significant (p=0.37).
