# Supplementary material for: The Dilated Cardiomyopathy-Causing Mutation ACTC E361G in Cardiac Muscle Myofibrils Specifically Abolishes Modulation of Ca2+ Regulation by Phosphorylation of Troponin I
Source: Biophys J. 2014 Nov 18;107(10):2369–80. doi: 10.1016/j.bpj.2014.10.024 (PMC4241448; doi:10.1016/j.bpj.2014.10.024)
Supplement: Document S2. Article plus Supporting Material [file mmc2.pdf]

Biophysical Journal Volume 107 November 2014 2369–2380

Article

The Dilated Cardiomyopathy-Causing Mutation *ACTC* E361G in Cardiac Muscle Myofibrils Specifically Abolishes Modulation of Ca^{2+} Regulation by Phosphorylation of Troponin I

Petr G. Vikhorev,[1] Weihua Song,[2] Ross Wilkinson,[1] O'Neal Copeland,[1] Andrew E. Messer,[1] Michael A. Ferenczi,[2] and Steven B. Marston[1,*]
[1]National Heart and Lung Institute, Imperial College London, London, UK; and [2]Lee Kong Chian School of Medicine, Nanyang Technological University, Singapore

ABSTRACT Phosphorylation of troponin I by protein kinase A (PKA) reduces Ca^{2+} sensitivity and increases the rate of Ca^{2+} release from troponin C and the rate of relaxation in cardiac muscle. In vitro experiments indicate that mutations that cause dilated cardiomyopathy (DCM) uncouple this modulation, but this has not been demonstrated in an intact contractile system. Using a Ca^{2+}-jump protocol, we measured the effect of the DCM-causing mutation *ACTC* E361G on the equilibrium and kinetic parameters of Ca^{2+} regulation of contractility in single transgenic mouse heart myofibrils. We used propranolol treatment of mice to reduce the level of troponin I and myosin binding protein C (MyBP-C) phosphorylation in their hearts before isolating the myofibrils. In nontransgenic mouse myofibrils, the Ca^{2+} sensitivity of force was increased, the fast relaxation phase rate constant, k_{REL}, was reduced, and the length of the slow linear phase, t_{LIN}, was increased when the troponin I phosphorylation level was reduced from 1.02 to 0.3 molPi/TnI (EC_{50} P/unP $= 1.8 \pm 0.2$, $p < 0.001$). Native myofibrils from *ACTC* E361G transgenic mice had a 2.4-fold higher Ca^{2+} sensitivity than nontransgenic mouse myofibrils. Strikingly, the Ca^{2+} sensitivity and relaxation parameters of *ACTC* E361G myofibrils did not depend on the troponin I phosphorylation level (EC_{50} P/unP $= 0.88 \pm 0.17$, $p = 0.39$). Nevertheless, modulation of the Ca^{2+} sensitivity of *ACTC* E361G myofibrils by sarcomere length or EMD57033 was indistinguishable from that of nontransgenic myofibrils. Overall, EC_{50} measured in different conditions varied over a 7-fold range. The time course of relaxation, as defined by t_{LIN} and k_{REL}, was correlated with EC_{50} but varied by just 2.7- and 3.3-fold, respectively. Our results confirm that troponin I phosphorylation specifically alters the Ca^{2+} sensitivity of isometric tension and the time course of relaxation in cardiac muscle myofibrils. Moreover, the DCM-causing mutation *ACTC* E361G blunts this phosphorylation-dependent response without affecting other parameters of contraction, including length-dependent activation and the response to EMD57033.

INTRODUCTION

The heart pumps blood around the body due to the contraction of heart muscle cells. The molecular motor of contraction is the interaction between myosin cross-bridges in the thick filaments and actin in the thin filaments, and is powered by hydrolysis of ATP in the myosin cross-bridge. The rhythmic contraction and relaxation is controlled by varying the intracellular Ca^{2+} concentration, which acts by binding and dissociating from troponin C, the Ca^{2+}-sensing component of the cooperative troponin/tropomyosin switch that is an integral component of the thin filaments (1).

In normal human heart, this all-or-nothing Ca^{2+}-dependent control mode is modulated by the activation of the sympathetic nervous system when demand for cardiac output increases. β-adrenergic stimulation mainly acts via activation of protein kinase A (PKA) by cyclic AMP and leads to an increased heart rate (chronotropy), increased force of contraction (inotropy), and increased rate of relaxation (lusitropy) (2,3).

PKA phosphorylates several key intracellular regulatory proteins, including myosin binding protein C (MyBP-C) and troponin I within the myofibril. Early studies showed that β-adrenergic stimulation of contraction is associated with enhanced phosphorylation of troponin I (4,5), troponin I is bisphosphorylated at Ser-22 and Ser-23 in the cardiac-specific N-terminal extension by PKA (6–8), and the primary effect of phosphorylation of troponin I in vitro is reduced Ca^{2+} sensitivity and faster dissociation of Ca^{2+} from troponin C (5,9,10). This can cause an increase in the rate of relaxation (the lusitropic response), which is essential when the heart rate is increased (3,11). Transgenic mouse studies have demonstrated the physiological importance of troponin I phosphorylation, as mice with unphosphorylatable troponin I have a blunted response to β-adrenergic stimulation that leads to an enhanced susceptibility to develop heart failure under stress (12–14).

Submitted April 25, 2014, and accepted for publication October 16, 2014.

*Correspondence: s.marston@imperial.ac.uk

This is an open access article under the CC BY license (http://creativecommons.org/licenses/by/3.0/).

Editor: Bernhard Brenner.

© 2014 The Authors

0006-3495/14/11/2369/12 $2.00

CrossMark

http://dx.doi.org/10.1016/j.bpj.2014.10.024

Dilated cardiomyopathy (DCM) is a major cause of heart failure in humans, and a substantial proportion of cases of DCM are inherited. Mutations in the thin-filament proteins (actin, tropomyosin, troponin T, troponin I, and troponin C) that are associated with familial DCM have been studied particularly closely (15–17). By studying isolated thin filaments using a quantitative in vitro motility assay, Memo et al. (18) showed that in all of these DCM-causing mutations, the myofilament Ca^{2+} sensitivity is independent of the level of troponin I phosphorylation; therefore, by analogy to S22/23A transgenic mice, they proposed that this uncoupling was necessary and sufficient to cause the DCM phenotype. It is uncertain whether uncoupling is also present in intact muscles that produce force, although indirect evidence from the *ACTC* E361G DCM mouse indicated a blunted response to β-adrenergic stimulation that was compatible with the uncoupling of the relationship between Ca^{2+} sensitivity and troponin I phosphorylation observed in vitro (19,20). In this study, we examined the relationships between troponin I phosphorylation and Ca^{2+} regulation of contractility in single myofibrils from a mouse model of familial DCM (*ACTC* E361G) in comparison with wild-type mice. We measured the effects of changing the [Ca^{2+}], troponin I phosphorylation level, and sarcomere length (SL) on the isometric tension and relaxation rate after rapid Ca^{2+} jumps.

Recent studies have shown that Ca^{2+} sensitivity decreased 2- to 3-fold between 0% and ~70% bisphosphorylation of troponin I and did not change at higher levels of phosphorylation (18,21). Since native mouse and human donor heart preparations have phosphorylation levels in the 50–70% range (22), the major effects of phosphorylation would be observed if phosphorylation levels were reduced rather than increased above normal, which is the more usual experimental situation (23). Therefore, we used propranolol treatment of mice to reduce the level of troponin I and MyBP-C phosphorylation in their hearts before isolating the myofibrils. Our results confirm that phosphorylation specifically alters the Ca^{2+} sensitivity of isometric tension and the time course of relaxation in wild-type myofibrils. Moreover, the DCM-causing mutation *ACTC* E361G blunts this phosphorylation-dependent response, as predicted by the in vitro motility studies, without affecting other parameters of contraction, including length-dependent activation (LDA) and the response to the Ca^{2+} sensitizer EMD57033.

MATERIALS AND METHODS

We used heart muscle from heterozygous *ACTC* E361G transgenic mice (previously described by Song et al. (24)) and nontransgenic (NTG) mice (hybrid strain C57Bl/6xCBA/Ca) as controls (male and female, 4–28 weeks old). Experiments and animal handling were done in accordance with the guidelines of the Imperial College London. Mice were killed by cervical dislocation as required by Schedule I of the UK Animals (Scientific Procedures) Act 1986.

Propranolol treatment

Mice were anesthetized with 5% isoflurane (IsoFlo, Abbott Laboratories, Berkshire, UK) v/v in 100% oxygen (0.5mL/min), weighed, and then transferred to a heated surgical table (VetTech, UK) where anesthesia was maintained at 2.5% isoflurane v/v in 100% O_2 (0.5 mL/min) using a custom-made nose cone. A bolus of propranolol (8 mg/kg BW; Sigma-Aldrich, Poole, UK) was injected into the subclavian vein. The mice were kept in an anesthesia induction chamber for 30 min with 1.5% isoflurane and then sacrificed. The heart was removed and tissue samples (<5 mg) were dissected from the left ventricle, frozen, and kept in liquid nitrogen until myofibrils were prepared. Control samples were obtained from anesthetized mice that did not receive propranolol.

Isolated myofibrils

Single myofibrils or thin bundles (two to seven myofibrils) were prepared from one piece of frozen heart sample by permeabilization and subsequent homogenization. Samples were immersed for 3 h in 2 mL of permeabilization solution containing (mM) Tris 10 (pH 7.1), NaCl 132, KCl 5, $MgCl_2$ 1, EGTA 5, dithiothreitol (DTT) 5, NaN_3 10, 2,3-butanedione-monoxime (BDM) 20, and 1% Triton X-100. All of our solutions contained the following protease inhibitors (μM): chymostatin 10, pepstatin 5, leupeptin 10, *trans*-epoxysuccinyl-L-leucylamido(4-guanidino)butane) (E-64) 10, and phenylmethylsulfonyl fluoride (PMSF) 200. Triton X-100 and BDM were then removed from the permeabilized samples with a washing solution (like the permeabilization solution, but without Triton X-100 and BDM), and finally the samples were homogenized for 15 s with an Ultra-Turrax T10 blender (IKA Werke, Staufen, Germany) to produce a suspension of myofibrils. The suspension was washed two times by centrifugation and suspension in the washing solution. The final pellet was dissolved in 300 μL of washing solution and kept on ice for use within 3 days.

In total, we used eight hearts (two from NTG propranolol-treated mice (4 and 28 weeks old), two from NTG-untreated (4 and 28 weeks old), two from *ACTC* E361G propranolol-treated mice (15 weeks and 28 weeks old), and two from *ACTC* E361G-untreated mice (5 weeks and 28 weeks old)). Three to seven myofibrillar preparations were prepared for each experimental condition.

Apparatus for measurement of myofibril contractility

We initiated contraction and relaxation using a fast-solution-change system and sensitive force transducer system, which were similar to those described previously (25–28). Briefly, our apparatus for measuring force in single myofibrils was built around an inverted microscope (Eclipse Ti-U; Nikon UK, Surrey, UK) equipped with two micromanipulators (MP-285, Sutter Instruments, Novato, and a Huxley-type micromanipulator) and a CCD camera (Rolera XR; Qimaging, Surrey, Canada). The myofibrils were manipulated by means of two fine glass microneedles (one of which was a cantilever force sensor) mounted on the micromanipulators. Under illumination of a 5 mW HeNe laser, the shadow of the tip of the cantilever force sensor was projected on a photodiode position detector (Spot-2D; UDT Sensors, Hawthorne, CA). The extent of bending of the cantilever was proportional to the force on the cantilever, so the force produced by a myofibril was measured from the photodiode's current response. We calibrated each cantilever force sensor by measuring its compliance using the needle of a microammeter to apply known forces to the cantilever and observing the extent of bending; the range of measured cantilever compliances was 2–14 μm/μN.

We achieved rapid activation and relaxation in myofibrils using an ultrafast-solution-change system constructed from a double-barreled micropipette mounted on a stepper motor that switched solutions in less than 10 ms. The micropipette was positioned perpendicular to the long axis of

the myofibril. The relaxing and activating solutions were applied via adjacent barrels of the micropipette, with flow being driven by gravity. The stepper motor controlled the position of the micropipette relative to the myofibril, thus enabling changes between the two solutions, each flowing in a laminar pattern.

An eight-channel valve (HVXM 8-5; Hamilton, VWR, Lutterworth, UK) controlled by a stepper motor was used to perfuse one barrel of the micropipette with a range of different activating solutions. We applied eight different solutions ranging from low to high [Ca^{2+}] (Fig. 1). The time lag in a change of solutions was less than 5 s.

Experimental solutions

Activating (3.16–0.1 μM Ca^{2+}) and relaxing (0.01 μM Ca^{2+}) solutions contained (mM) MOPS 10 (pH 7.0), MgATP 5, free Mg^{2+} 1, DTT 5, phosphocreatine 10, and creatine kinase (200 U/mL). The Ca-EGTA/EGTA ratio was set to obtain 10 mM total EGTA and the desired free [Ca^{2+}]. Potassium propionate and sodium sulfate were added to adjust the ionic strength of the final solution to 200 mM. EMD57033 was dissolved in ethanol (30 mM) and then added in some experiments to activating and relaxing solutions at a concentration of 30 μM. Myofibrils were incubated in EMD57033-containing relaxing solution for 20–30 min at 17°C before measurements were started.

Experimental protocol

A small droplet of myofibril solution was placed on the bottom of the glass chamber (temperature controlled to 17°C) and then the chamber was filled with relaxing solution. The selected myofibril or thin bundle (1–4 μm in diameter, 25–100 μm in length) was positioned horizontally between the

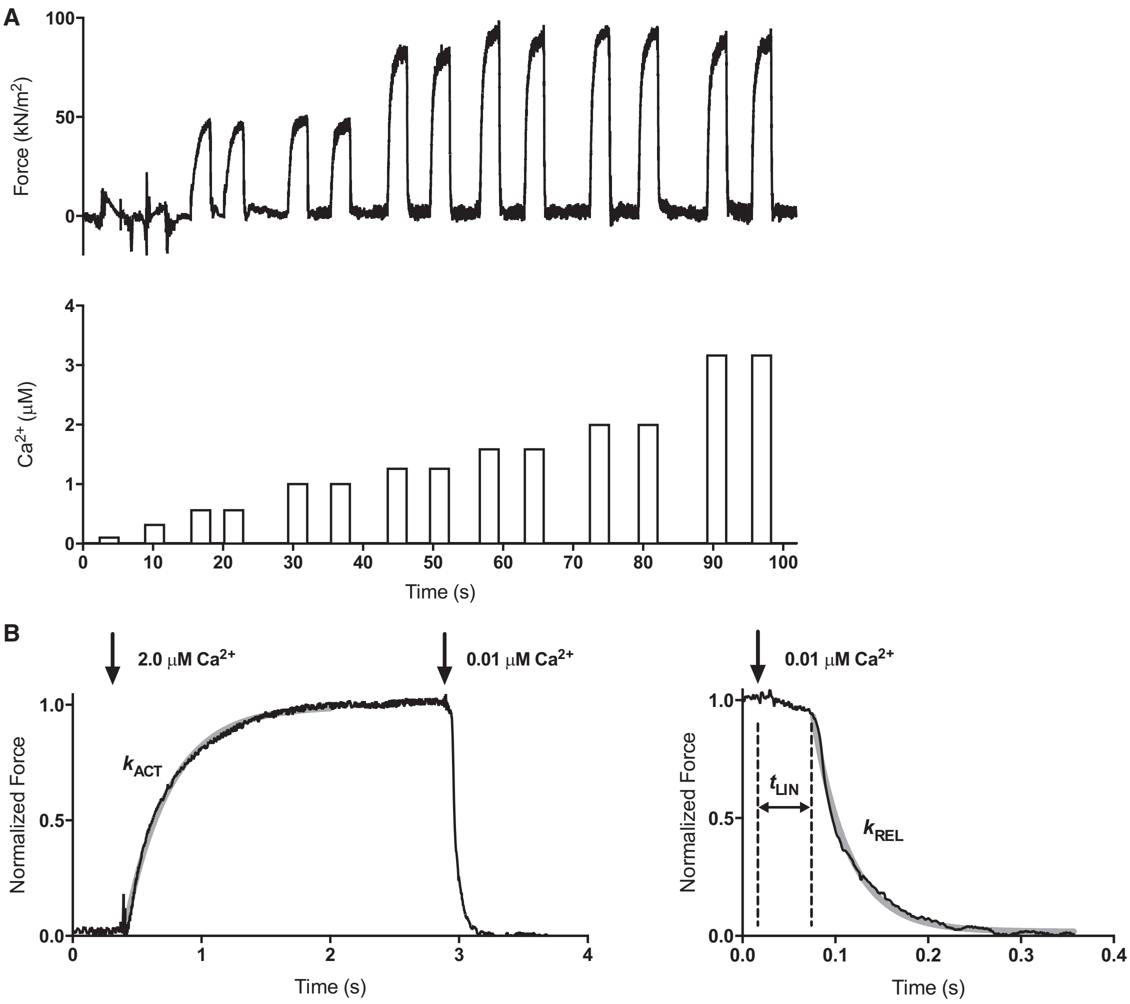

FIGURE 1 Measurement of contractility in single mouse cardiac muscle myofibrils. (*A*) A representative trace shows the contractile responses of the cardiac myofibril to different activating Ca^{2+} concentrations. Myofibrils were activated with increasing Ca^{2+} concentrations alternating with relaxing solution using a Ca^{2+}-jump protocol and various Ca^{2+}-activating solutions as indicated in the lower trace. Sometimes myofibrils were initially activated at high activating Ca^{2+} concentrations. (*B*) Time course of contraction and relaxation. The arrows indicate the time when the solutions were switched. The kinetic parameters maximum force (F_{max}), rate of force development (k_{ACT}), duration (t_{LIN}) of the slow relaxation phase, and rate of the fast relaxation phase (k_{REL}) were determined from the traces. The slow-relaxation phase was counted from the initiation of micropipette movement. The beginning of the exponential phase of relaxation was considered as the end of slow-relaxation phase. The relaxation phase is shown over an expanded timescale to show the initial linear, nearly isometric force decay period $t_{LIN,}$ followed by an exponential relaxation with a rate constant k_{REL}. The gray solid lines represent the best exponential fits to the data. Previous experiments (29) showed that $k_{ACT} = k_{TR}$, indicating the Ca^{2+} jump, was not rate limiting for $k_{ACT.}$ The temperature was 17°C.

microneedles described above and viewed with the video camera mounted on the microscope (Fig. S1 in the Supporting Material). Sarcomere length (SL) was set to 2.17 or 1.9 μm while observing the striation pattern in the myofibril image using the fast Fourier transform analysis function in LabVIEW. The length and diameter of the myofibril were measured using ImageJ (http://imagej.nih.gov/ij/). The cross-sectional area was calculated from the observed diameter assuming a circular cross-section. Contraction and relaxation were initiated by the fast-solution-switch system described above. At each [Ca^{2+}], a number of activation-relaxation cycles (range 1–5) were performed by the myofibril and the average value of measured parameters for the set of cycles was reported. Each contraction lasted for 2–5 s. If the myofibril sarcomere appearance or signal did not deteriorate after a series of contractions at short SL, the same myofibril was also used for measurements at long SL.

Data collection and analysis

Apparatus control and data recording were done using a data acquisition device (NI USB-6251; National Instruments, Newbury, UK) and custom-written software in LabVIEW 2011 (National Instruments). The rate constants for exponential force development upon activation (k_{ACT}) and fast phase of relaxation (k_{REL}) were evaluated by curve-fitting using the Levenberg-Marquardt nonlinear least-square algorithm in LabVIEW.

The maximum force was obtained by curve-fitting force development upon activation. The force data from the force-[Ca^{2+}] experiments were fitted to the Hill equation: $y = y_0 + F_{max}[Ca^{2+}]^{nH}/(EC_{50}{}^{nH} + [Ca^{2+}]^{nH})$ by adjusting the values of nH and EC_{50}. Data were fitted and compared by means of an unpaired t-test using GraphPad Prism 6 (GraphPad Software, San Diego, CA). Best-fit parameters marked as ambiguous by GraphPad Prism were excluded from further analysis. In some cases, the bottom plateau (y_0) was constrained to a constant value of 0.

Additionally, a paired t-test was also used to compare EC_{50} and F_{max} values at short and long SLs. Table 1 reports the means and SE of EC_{50}, nH, F_{max}, k_{ACT}, t_{LIN}, and k_{REL} values at maximum activation, as determined in six to 16 experiments with separate myofibrils. During the slow-relaxation phase, active tension essentially did not change, which made it difficult to accurately determine the rate constant k_{LIN} for the slow-relaxation phase. k_{LIN} was not significantly affected by phosphorylation, changes in SL, EMD57033, or the mutation *ACTC* E361G (mean values range from 0.20 to 0.51 s^{-1}), and thus is not discussed here (more information is provided in Fig. S2 and Fig. S5). For the illustrations, the Ca^{2+}-concentration plots show the mean and SE values of the parameter studied at each Ca^{2+} concentration and the curve fitted of the mean data points.

RESULTS

Propranolol treatment dephosphorylates mouse heart myofibrils

We studied myofibrils from NTG C57Bl/6xCBA/Ca mice and transgenic mice expressing 50% of their heart actin as the DCM-causing *ACTC* E361G mutation. NTG and mutant mice had similar levels of troponin I and MyBP-C phosphorylation. We found that after treatment with propranolol, the phosphorylation levels of both troponin I and MyBP-C, particularly the physiologically relevant bisphosphorylated species, were substantially reduced (Fig. 2). The contractilities of NTG and *ACTC* E361G phosphorylated and dephosphorylated myofibrils were compared.

MyBP-C and troponin I phosphorylation modulate myofibril contractility

We measured contraction-relaxation cycles in myofibrils with graded increases in [Ca^{2+}] (Fig. 1). The Ca^{2+} sensitivity of isometric force in the unphosphorylated myofibrils was always greater than that of the phosphorylated

TABLE 1 Summary values for Ca^{2+}-regulated myofibril contraction: effects of phosphorylation, sarcomere length, *ACTC* E361G mutation, and EMD57033

	SL		F_{max}, kN/m^2	EC_{50}, μM	nH	k_{ACT}, s^{-1}	t_{LIN}, ms	k_{REL}, s^{-1}
NTG	2.17	P	$100.9 \pm 6.3(14)$	$0.93 \pm 0.06(11)$	$10.43 \pm 1.84(10)$	$4.16 \pm 0.43(11)$	$50.8 \pm 3.5(12)$	$35.0 \pm 4.0(10)$
		unP	$87.1 \pm 6.0(16)$	$0.51 \pm 0.06(14)^{\dagger\dagger\dagger}$	$4.74 \pm 0.66(13)^{\dagger\dagger}$	$4.50 \pm 0.24(15)$	$67.0 \pm 4.2(16)^{\dagger}$	$23.2 \pm 2.8(11)^{\dagger\dagger\dagger}$
		P+EMD	$73.0 \pm 10.1(6)^{\S}$	$0.29 \pm 0.06(7)^{\S\S\S}$	$3.96 \pm 1.04(7)^{\S}$	$4.25 \pm 0.31(8)$	$125.8 \pm 16.8(7)^{\S\S\S}$	$12.8 \pm 2.2(6)^{\S\S\S}$
		unP+EMD	$87.0 \pm 9.0(8)$	$0.17 \pm 0.05(11)^{\S\S\S}$	$3.96 \pm 0.98(10)$	$4.61 \pm 0.65(9)$	$103.9 \pm 8.4(8)^{\S\S\S}$	$10.7 \pm 2.1(8)^{\S\S}$
	1.9	P	$71.0 \pm 6.4(7)^{\ddagger\ddagger}$	$1.18 \pm 0.16(7)$	$5.17 \pm 0.71(7)^{\ddagger}$	$4.26 \pm 0.44(7)$	$50.0 \pm 7.1(7)$	$31.9 \pm 5.2(6)$
		unP	$75.4 \pm 4.7(13)$	$0.55 \pm 0.06(12)^{\dagger\dagger\dagger}$	$4.85 \pm 1.12(9)$	$4.32 \pm 0.44(12)$	$68.4 \pm 6.9(11)^{\dagger}$	$27.2 \pm 3.6(10)$
		P+EMD	$73.3 \pm 4.9(11)$	$0.35 \pm 0.06(8)^{\S\S\S}$	$6.43 \pm 2.12(7)$	$4.89 \pm 0.40(12)$	$92.1 \pm 11.8(9)^{\S\S}$	$18.6 \pm 3.3(8)^{\S\S}$
		unP+EMD	$87.9 \pm 8.2(9)$	$0.30 \pm 0.07(10)^{\S}$	$2.48 \pm 0.71(9)$	$4.71 \pm 0.25(12)$	$102.5 \pm 9.4(10)$	$12.5 \pm 2.3(8)^{\S\S}$
ACTC E361G	2.17	P	$93.5 \pm 8.9(11)$	$0.38 \pm 0.05\ (12)^{***}$	$5.39 \pm 1.26(9)^{*}$	$4.51 \pm 0.32(13)$	$75.4 \pm 6.5\ (10)^{**}$	$21.6 \pm 2.8\ (10)^{***}$
		unP	$89.3 \pm 8.6(10)$	$0.43 \pm 0.06\ (10)$	$4.48 \pm 0.70(8)$	$4.12 \pm 0.32(11)$	$70.8 \pm 9.7(11)$	$21.6 \pm 2.1(10)$
		unP+EMD	$90.0 \pm 7.2(8)$	$0.21 \pm 0.05\ (8)^{\S\S\S}$	$2.73 \pm 0.93(7)$	$3.66 \pm 0.30(8)$	$135.6 \pm 25.0(7)^{\S}$	$12.5 \pm 2.6(7)^{\S}$
	1.9	P	$87.3 \pm 9.2(10)$	$0.66 \pm 0.18\ (6)$	$5.78 \pm 1.60(6)$	$4.86 \pm 0.37\ (9)$	$71.3 \pm 8.6(7)$	$29.8 \pm 3.0\ (7)$
		unP	$71.1 \pm 6.8(7)$	$0.74 \pm 0.11\ (7)^{\dagger\ddagger}$	$7.52 \pm 2.28(6)$	$3.55 \pm 0.49(6)$	$60.0 \pm 8.9(6)$	$26.8 \pm 5.9(6)$
		unP+EMD	$78.1 \pm 5.1(9)$	$0.22 \pm 0.03(9)^{\S\S\S}$	$4.31 \pm 0.83(8)$	$4.40 \pm 0.18(11)$	$106.8 \pm 15.6(8)^{\S}$	$13.9 \pm 5.2(8)$

The maximal developed force (F_{max}), concentration of Ca^{2+} for half-maximal force (EC_{50}), and Hill coefficient (nH) were estimated by fitting the Hill equation to the data from single experiments. F_{max} was normalized to the myofibril cross-section area. Activation and relaxation rate constants (k_{ACT} and k_{REL}, respectively) were determined from the best fit to an exponential equation. The time delay for the fast exponential relaxation after the solution switch is shown as t_{LIN}. Each data point is the average value $\pm$ SE for the measurements obtained on different myofibrils (shown in parentheses). P, phosphorylated myofibrils; unP, unphosphorylated myofibrils; EMD, EMD57033. The temperature was 17°C.

$^{*}p < 0.05$, $^{**}p < 0.01$, $^{***}p < 0.001$; *ACTC* E361G compared with NTG.

$^{\dagger}p < 0.05$, $^{\dagger\dagger}p < 0.01$, $^{\dagger\dagger\dagger}p < 0.001$; unphosphorylated compared with phosphorylated.

$^{\ddagger}p < 0.05$.$^{\ddagger\ddagger}p < 0.01$; 1.9 μm SL compared with 2.17 μm SL.

$^{\S}p < 0.05$, $^{\S\S}p < 0.01$, $^{\S\S\S}p < 0.001$; EMD57033 incubated compared with no EMD57033.

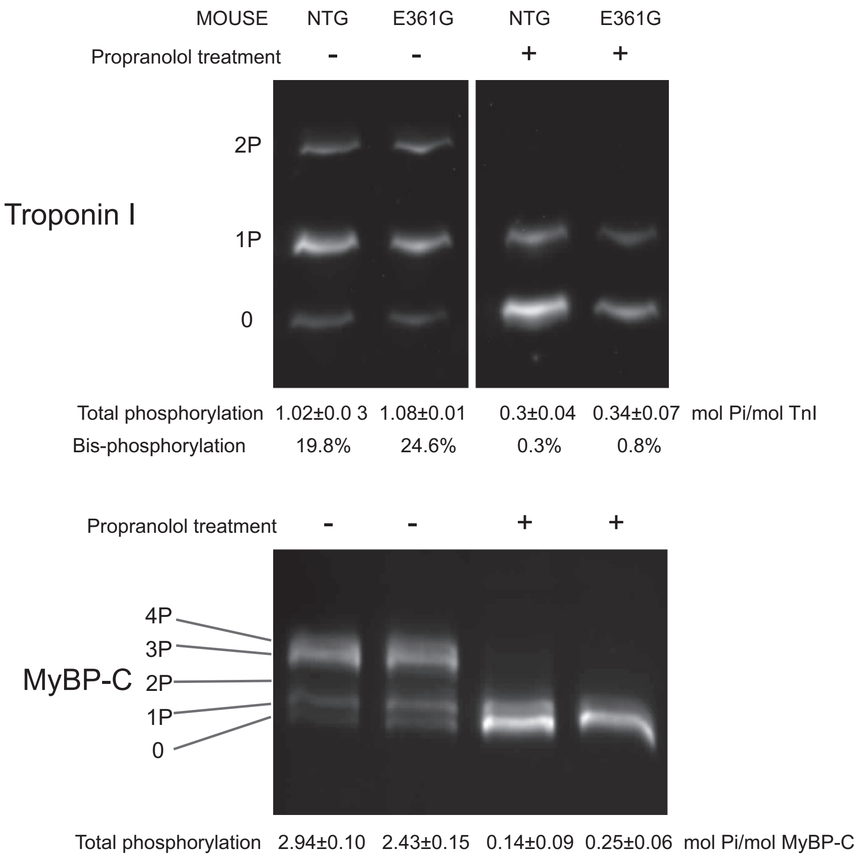

FIGURE 2 Dephosphorylation of troponin I and MyBP-C in mouse heart after propranolol treatment. Phosphorylation of troponin I and MyBP-C in NTG and *ACTC* E361G mice was measured by phosphate-affinity SDS-PAGE. Differently phosphorylated protein forms were separated by the number of phosphate groups: 0P, nonphosphorylated; 1P, monophosphorylated; 2P, diphosphorylated; 3P, triphosphorylated; 4P, tetraphosphorylated. Three different gels were prepared for each sample and the mean with SE is shown.

myofibrils (EC_{50} P/unP $= 2.1 \pm 0.4\times$ at 1.9 μm SL, $p < 0.001$; and $1.8 \pm 0.2\times$ at 2.17 SL, $p < 0.001$; Fig. 3; Table 1). The Hill coefficient was greater in phosphorylated compared with unphosphorylated myofibrils (2.2 ± 0.5 fold, $p = 0.007$). The shift in Ca^{2+} sensitivity is the same as that observed in isolated mouse thin filaments measured by the in vitro motility assay (EC_{50} P/unP $= 2.0 \pm 0.2$ (18)).

The rate constant for contraction, k_{ACT}, was not affected by phosphorylation, but changes in the relaxation parameters indicated that relaxation was faster in the phosphorylated myofibrils (t_{LIN} phosphorylated myofibrils $= 50.8 \pm 3.5$ ms, unphosphorylated myofibrils $= 67.0 \pm 4.2$ ms, $p = 0.015$; k_{REL} phosphorylated myofibrils $= 35.0 \pm 4.0$ s^{-1}, unphosphorylated myofibrils $= 23.2 \pm 2.8$ s^{-1}, $p < 0.001$ at 2.17 μm SL; see Table 1). The difference in relaxation rate parameters between phosphorylated and unphosphorylated NTG myofibrils was less pronounced at short SLs.

Effect of the *ACTC* E361G mutation on myofibril contractility

At both long and short SLs, native phosphorylated *ACTC* E361G mouse myofibrils were activated at lower Ca^{2+} concentrations than NTG myofibrils. For example, EC_{50} of *ACTC* E361G myofibrils at 2.17 μm SL $= 0.38 \pm 0.05$ μM, compared with 0.93 ± 0.06 μM for the NTG mouse myofibrils ($p < 0.001$). Correspondingly, relaxation of *ACTC* E361G myofibrils was significantly slower than that of NTG myofibrils (longer t_{LIN} and slower k_{REL} compared with NTG; Fig. 4; Table 1). The Hill coefficient was lower for *ACTC* E361G mice (5.39 ± 1.26 vs. 10.43 ± 1.84, $p < 0.05$). There was no significant difference in the maximum force produced or the rate of force development (Table 1).

The *ACTC* E361G mutation uncouples phosphorylation-dependent changes in contractile parameters

In contrast to NTG mouse myofibrils, the Ca^{2+} sensitivity was the same independently of the phosphorylation level (EC_{50} for phosphorylated *ACTC* E361G $= 0.38 \pm 0.05$ μM, and for unphosphorylated $= 0.43 \pm 0.06$ μM (2.17 μm SL); $p = 0.394$). Similarly, there was no significant phosphorylation-dependent change in the time course of relaxation at fully activating $[Ca^{2+}]$ ($p = 0.703$ (t_{LIN}), $p = 0.912$ (k_{REL}); Fig. 5). The uncoupling observed in myofibrils corresponds to the uncoupling previously identified in isolated thin filaments carrying this mutation (18,24). On the other hand, modulation of Ca^{2+} sensitivity in *ACTC* E361G myofibrils by a change in SL or addition of the Ca^{2+} sensitizer EMD57033 was

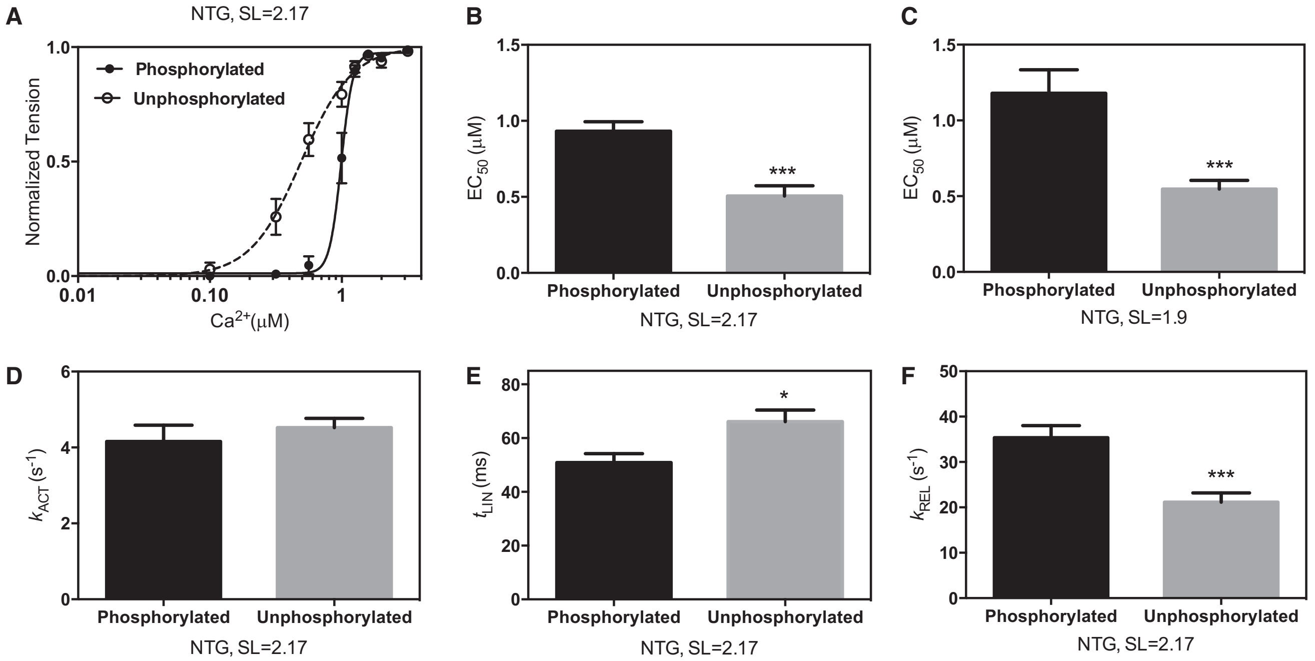

FIGURE 3 Effect of dephosphorylation on mouse myofibril contractility. (*A*) The Ca^{2+} force sensitivity curve for unphosphorylated myofibrils (*open circles*, *dashed line*) is shifted to the left of the phosphorylated myofibrils (*solid circles*, *solid line*). The plot shows averaged data from 11–14 myofibrils for experiments performed at SL = 2.17 μm. (*B* and *C*) Means of calculated Ca^{2+} concentrations for the half-maximum force response (EC_{50}) at SL of 2.17 μm (*B*) and 1.9 μm (*C*). (*D–F*) Kinetic parameters k_{ACT}, t_{LIN}, and k_{REL} at maximally activating Ca^{2+} and SL = 2.17 μm. Values are means ± SE. *$p < 0.05$, ***$p < 0.001$. Data from Table 1.

similar to that observed for NTG myofibrils (Table 1; Figs. 6 and S3).

Effect of the Ca^{2+} sensitizer EMD57033 on myofibril contractility

We found that 30 μM EMD57033 increased the myofibrillar Ca^{2+} sensitivity by 1.8- to 3.4-fold and the maximum force by 0–17% (Table 1). EMD57033 also slowed relaxation, but did not affect k_{ACT} significantly (4.16 ± 0.43 s^{-1} and 4.25 ± 0.31 s^{-1} in the absence and presence of EMD57033, respectively; $p = 0.868$, NTG SL = 2.17). Interestingly, the Ca^{2+}-sensitizing effect was independent of the myofibril phosphorylation level, the SL, and the presence of the *ACTC* E361G mutation (Fig. S3; Table 1).

Since EMD57033 strongly decreases the Ca^{2+} level required to initiate contraction, we used the myosin inhibitor BDM in two additional experiments to ensure that the myofibrils were fully relaxed in the relaxing solution. At 20 s after a regular contraction-relaxation, a second relaxing solution with 40 mM BDM and without EMD57033 was applied to the myofibrils for 10 s. In these experiments, we used *ACTC* E361G dephosphorylated myofibrils. We did not see any changes in the force level. Thus, BDM did not increase myofibrillar relaxation further or such changes were less than 3% of the maximum force and could not be seen because of the signal fluctuations.

Effect of SL change on Ca^{2+} regulation

Measurements of Ca^{2+} activation in NTG myofibrils at 1.9 and 2.17 μm SL demonstrated LDA, with an increase in Ca^{2+} sensitivity of 1.24- to 1.32-fold after stretch, in phosphorylated and unphosphorylated myofibrils. The maximum developed force increased by 42% and 29% in phosphorylated and unphosphorylated myofibrils, respectively, upon stretch. t_{LIN} increased and k_{REL} decreased upon stretch, indicating a slower relaxation rate (Table 1; Fig. 6). LDA was not compromised by the *ACTC* E361G mutation or EMD57033 treatment.

DISCUSSION

We used the rapid Ca^{2+}-jump technique to investigate the equilibria and kinetics of Ca^{2+}-regulation of mouse heart myofibrils when it is modulated by PKA-dependent phosphorylation of contractile proteins. We found that a DCM-causing mutation in actin (*ACTC* E361G) specifically interferes with this modulation of Ca^{2+} sensitivity.

Modulation of Ca^{2+} sensitivity by troponin I phosphorylation

The troponin I of NTG mice hearts was phosphorylated at a level of 1.02 ± 0.03 mol Pi/mol (20% bisphosphorylation at

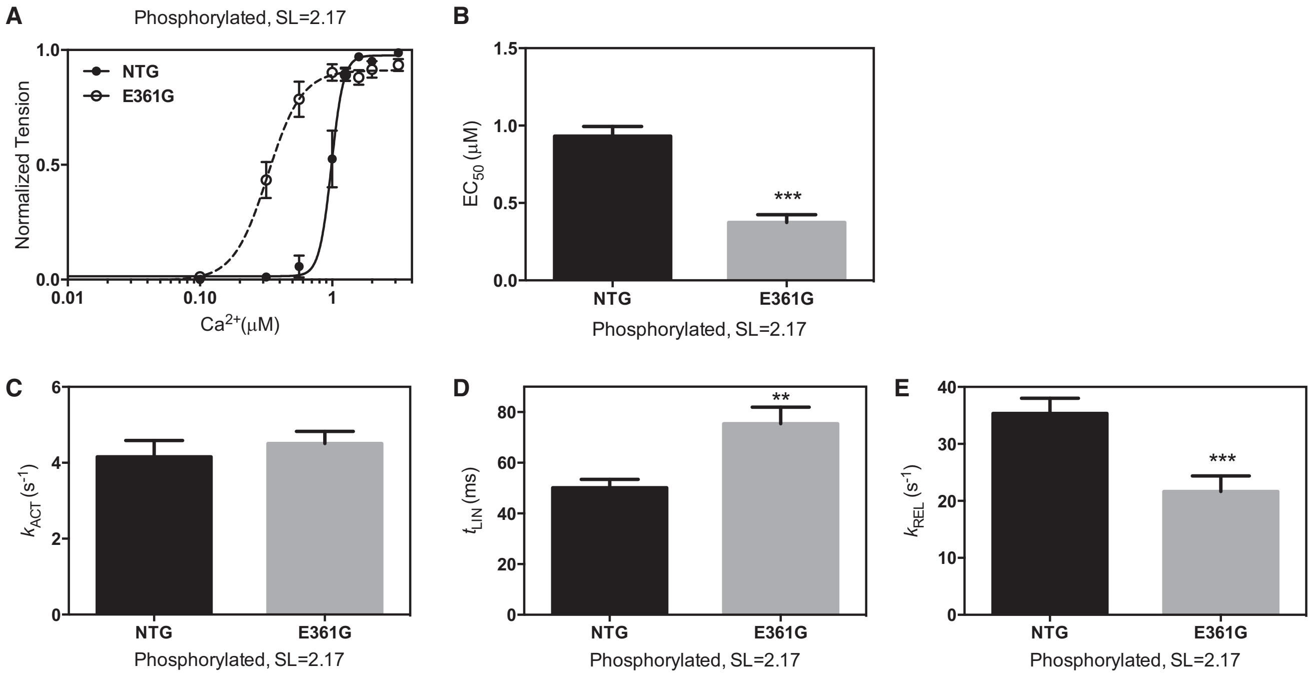

FIGURE 4 Effect of the *ACTC* E361G DCM-causing mutation on mouse myofibril contractility. (*A*) Force-Ca^{2+} relationship for NTG (*solid circles, solid line*) and *ACTC* E361G (*open circles, dashed line*) at SL = 2.17 μm. (*B*) EC_{50} was lower in *ACTC* E361G myofibrils. (*C–E*) Kinetic parameters of force development and relaxation (k_{ACT}, t_{LIN}, and k_{REL}, respectively). (*D* and *E*) *ACTC* E361G myofibrils showed a longer duration of the initial linear slow phase (t_{LIN}) of relaxation (*D*) and a slower rate of the subsequent fast exponential tension decay (*E*). Values are means ± SE. $^{**}p < 0.01$, $^{***}p < 0.001$. Data from Table 1.

Ser-22/23) in common with previous measurements (22,24). We achieved substantial in vivo dephosphorylation of troponin I and MyBP-C by treating the mice with propranolol before extracting the heart and preparing myofibrils (Fig. 2) (30). Total phosphorylation levels were reduced to 1/3 and physiologically relevant bisphosphorylation was reduced to less than 1/20.

Several studies have indicated that the major change in Ca^{2+} sensitivity occurs over the range of 0–70% occupancy of Ser-22 and Ser-23, with a relatively small decrease in Ca^{2+} sensitivity at higher levels of phosphorylation of troponin I (4,18,21). The basal level of phosphorylation in mouse and human donor heart samples is in the range of 50–75% occupancy and there is considerable uncertainty as to whether this is equal to or greater than the normal in vivo phosphorylation level (31,32). Since the low phosphorylation range could be accessed by β-blocker treatment of whole mouse, it is likely that it represents a physiologically relevant range.

An increase in Ca^{2+} sensitivity of isometric force was observed in NTG mouse myofibrils after dephosphorylation, and its magnitude was similar to that reported for single thin filaments by in vitro motility assay (18,24) and in skinned heart muscle fibers and myofibrils (10,23,33). The Hill coefficient was significantly smaller in unphosphorylated myofibrils. Changes in the phosphorylation level of troponin I did not affect the maximum force but did increase the relaxation rate at maximally activating [Ca^{2+}], in accord with previous observations in skinned cardiac fibers (10,11). The phosphorylation-dependent increase in the rates of relaxation that accompanies decreased Ca^{2+} sensitivity (Fig. 3) may directly contribute to the lusitropic effect of troponin I phosphorylation in addition to its likely effect on sarcoplasmic Ca^{2+} buffering (34).

It should be noted that propranolol treatment dephosphorylates both troponin I and MyBP-C, but the effects on Ca^{2+} regulation observed here reflect only the known properties of troponin I phosphorylation. MyBP-C phosphorylation has been shown to modulate the rate of stretch activation (35); however, all of the measurements presented here are isometric. It is interesting to note that steady-state LDA was not significantly affected by the level of troponin I phosphorylation, being 1.24× in phosphorylated and 1.32× in unphosphorylated myofibrils. The impact of phosphorylation on LDA has not been extensively studied, but Rao et al. (36) reported similar LDA values in native (phosphorylated) and S23/24A exchanged (unphosphorylatable) mouse heart fibers. They also reported that after PKA treatment of NTG, S23/24A-exchanged, or S23/24D (pseudophosphorylated) heart fibers, LDA became negligible. Thus, it seems that supraphysiological phosphorylation, presumably of some species other than troponin I, may be detrimental to LDA.

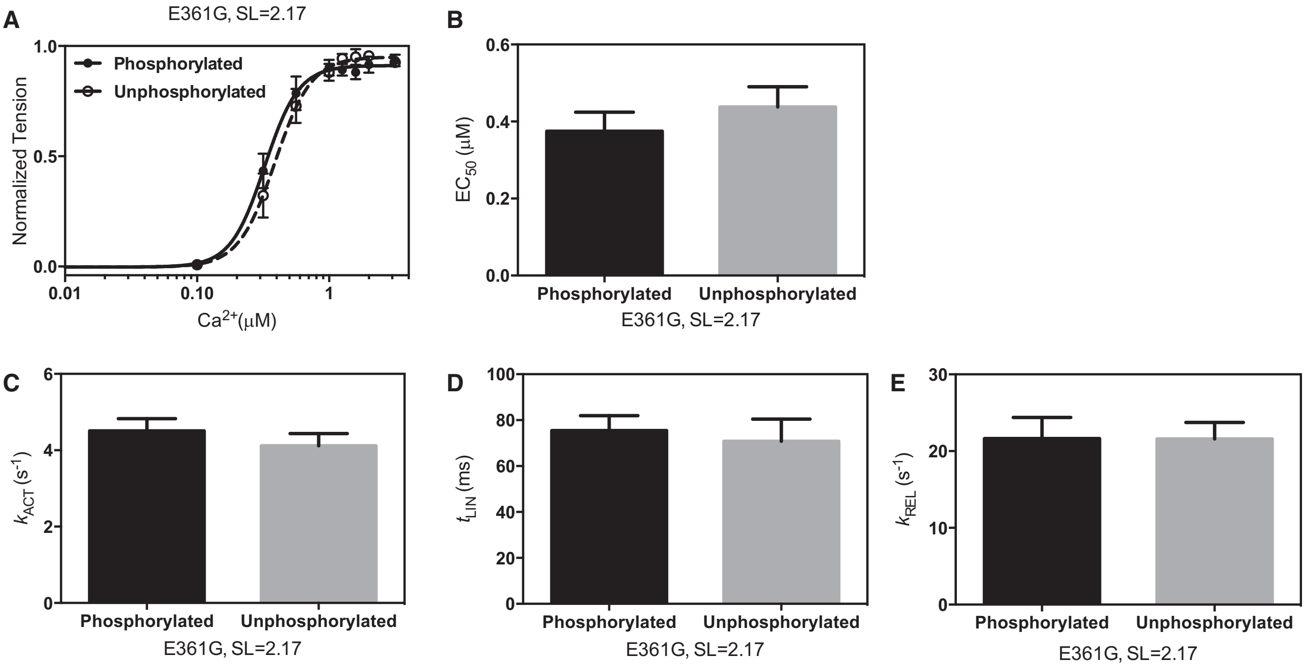

FIGURE 5 Effect of dephosphorylation on *ACTC* E361G mouse myofibril contractility. (*A* and *B*) Force-Ca^{2+} relationship for phosphorylated (*solid circles*, *solid line*) and unphosphorylated (*open circles*, *dashed line*) *ACTC* E361G myofibrils (*A*) and the corresponding mean EC_{50} values ($n = 10$–12) (*B*). (*C*–*E*) Kinetic parameters of force development and relaxation (k_{ACT}, t_{LIN}, and k_{REL}, respectively). SL = 2.17. Values are means ± SE. Data from Table 1.

The DCM-causing mutation *ACTC* E361G uncouples troponin I phosphorylation from changes in Ca^{2+} sensitivity and relaxation rate

When we studied myofibrils from the *ACTC* E361G transgenic mouse model of DCM, we found that the Ca^{2+} sensitivity of isometric force was not dependent on troponin I phosphorylation; likewise, there was no significant difference in k_{ACT} or k_{REL} between natively phosphorylated and unphosphorylated myofibrils. This uncoupling effect of the *ACTC* E361G mutation was previously reported for isolated thin filaments by in vitro motility assay and confirms that uncoupling also occurs in the fully assembled myofibril. We previously reported that many other mutations associated with DCM uncouple the phosphorylation dependence of Ca^{2+} sensitivity (18). Interestingly, LDA and the Ca^{2+}-sensitizing effect of EMD57033 were not affected by the *ACTC* E361G mutation. Similarly, Inoue et al. (37) reported only a modest decrease in LDA (from

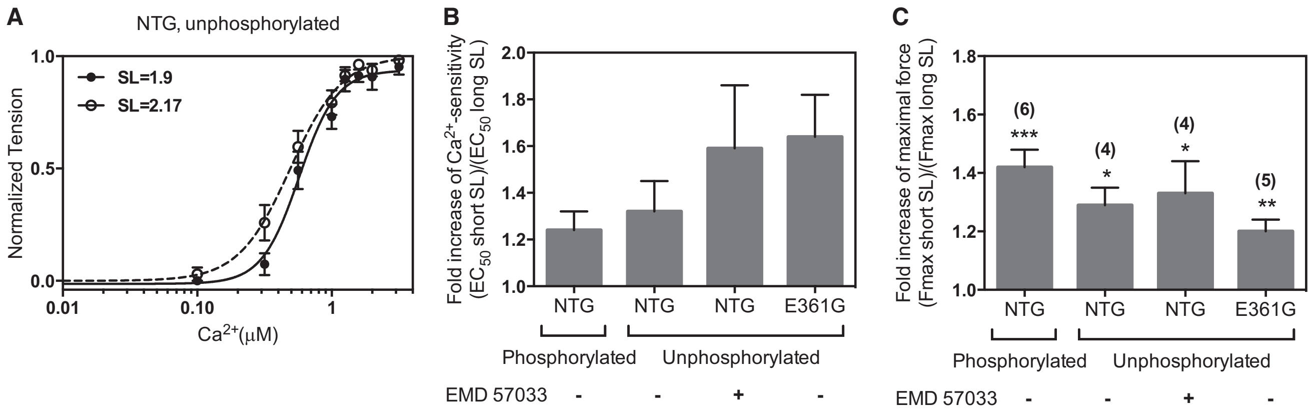

FIGURE 6 Length-dependent modulation of contraction in mouse cardiac myofibrils. (*A*) Force-Ca^{2+} relationship at SLs 2.17 μm (*open circles*, *dashed line*) and 1.9 (*solid circles*, *solid line*) for unphosphorylated NTG myofibrils. (*B*) Ratio of EC_{50} at short SL to EC_{50} at long SL measured in the same myofibril ($n = 3$–5 in each experiment), indicating LDA. The *p*-values (paired *t*-test) for the four conditions are 0.058, 0.097, 0.091, and 0.070, respectively. (*C*) Ratio of F_{max} at short SL to F_{max} at long SL measured in the same myofibril. Mean ratios ± SE under each condition are shown. The number of measurements (*n*) is shown in parentheses. $^{*}p < 0.05$, $^{**}p < 0.01$, $^{***}p < 0.001$.

1.6× to 1.4×) in heterozygous *TNNT2* ΔK210 KI mice compared with NTG mice.

The observation of uncoupling in myofibrils is compatible with the hypothesis that a specific interaction between the N-terminus of cardiac troponin I (residues 1-30) and troponin C is responsible for the higher Ca^{2+} sensitivity of unphosphorylated troponin (38–40). We proposed that this interaction is uniquely unstable and can be disrupted by phosphorylation at Ser-22/23 or by mutations and other perturbations of the myofilaments (18,41). Modulation of troponin function by phosphorylation of the N-terminal extension of troponin I is unique to cardiac muscle and appears to be structurally and functionally independent of Ca^{2+} switching (42,43).

Compared with myofibrils from NTG mice, those from *ACTC* E361G mice had a substantially higher Ca^{2+} sensitivity of isometric tension (Fig. 4). In other systems, this mutation also increased Ca^{2+} sensitivity, although the magnitude of the difference was quite small (1.05-fold for in vitro motility assay of single thin filaments and 1.28-fold for skinned papillary muscle), whereas with skeletal muscle troponin, *ACTC* E361G decreased Ca^{2+} sensitivity (24). For several DCM mutations, including *ACTC* E361G, it has been shown that the effect of a mutation on Ca^{2+} sensitivity is variable and depends on the measurement conditions (see Table 2 of Memo et al. (18)). In addition, the hypothesis that Ca^{2+} sensitivity is always reduced by DCM-causing mutations is no longer tenable; other examples of DCM mutations that can increase Ca^{2+} sensitivity include *TNNC1* G159D (44), *TPM1* D230N (18), and *TPM1* E54K (45).

These results support our hypothesis that the primary abnormality in DCM-causing mutations in thin-filament proteins like *ACTC* E361G is uncoupling, and not a change in Ca^{2+} sensitivity. Uncoupling is likely to be of physiological importance for the development of the DCM phenotype. Abolition of the normal response to β-adrenergic stimulation is expected to reduce cardiac reserve and predispose to heart failure (13,14). We have demonstrated that in the whole *ACTC* E361G mouse, the response to the β1 agonist dobutamine is indeed strongly blunted (24) and the reduced cardiac reserve can lead to the development of heart failure under stress (20,46).

Ca^{2+} dependence of contraction and relaxation rates

Since we measured the kinetics of Ca^{2+}-activated contraction and relaxation over a wide range of EC_{50} values, it is instructive to consider the relationship between Ca^{2+} switching and rate constants. The overall pattern of results demonstrated three features: 1), Ca^{2+} regulates k_{ACT} and isometric tension, but not t_{LIN} or k_{REL}; 2), changes in Ca^{2+} sensitivity are related to changes in t_{LIN} or k_{REL}, but not k_{ACT}; and 3), modulation of Ca^{2+} sensitivity by LDA is unrelated to modulation of Ca^{2+} sensitivity by phosphorylation changes or the *ACTC* E361G mutation.

The rate of cross-bridge attachment is dependent on the fraction of switched-on troponin-tropomyosin units on the target thin filaments because this determines the probability of attachment. Thin-filament switching and cross-bridge recruitment are controlled by [Ca^{2+}]; therefore, k_{ACT} is expected to be Ca^{2+} dependent, as shown in Fig. 7 *A* and previously demonstrated for k_{ACT} and k_{TR} (11,26,47–50). In contrast, k_{REL} was found to be independent of [Ca^{2+}].

We observed that changes in the myofilament Ca^{2+} sensitivity, whether due to introducing a mutation, changing SL, changing the troponin I phosphorylation level, or adding EMD57033, were all associated with corresponding changes in the relaxation parameters at maximally activating [Ca^{2+}] (Fig. 7 *B*). The relationship is not direct, since a 7-fold change in EC_{50} led to only a 2.7-fold change in t_{LIN} and a 3.3-fold change in k_{REL}. In contrast, k_{ACT} was not correlated with EC_{50}. Previous measurements in murine myofibrils also showed a relationship between t_{LIN} and k_{REL} values and EC_{50} (50), whereas studies in skeletal muscle myofibrils and in human heart myofibrils did not (23,26).

Ca^{2+} sensitivity is determined by the affinity of Ca^{2+} for troponin C in thin filaments (45). The decrease in Ca^{2+} sensitivity observed when cardiac troponin I is phosphorylated is linked to an increase in the rate of Ca^{2+} dissociation from troponin C in thin filaments (9,51), and this provides a potential link between Ca^{2+} sensitivity and the rate of relaxation.

Relaxation is initiated by Ca^{2+} dissociating from troponin C coupled to the release of the C-terminus of troponin I from troponin C and its attachment to actin, where it blocks cross-bridge binding cooperatively (9,52). The lag in the force trace has been ascribed to the time taken for the occupancy of cycling cross-bridges to drop below the threshold for cooperative activation of the thin filament, and the subsequent rapid-relaxation phase corresponds to the detachment of the remaining cross-bridges, where reattachment is prevented by the Ca^{2+}-free troponin switch (53,54).

Little et al. (55) pointed out that although cross-bridge detachment is the slowest process in relaxation at low temperatures, the detachment rate increases strongly with temperature, whereas Ca^{2+} dissociation from troponin C does not; therefore, it is possible for Ca^{2+} dissociation to become partly rate limiting at higher temperatures. According to the model of Stehle et al. (49), the apparent rate of cross-bridge detachment, g, is given by the value of k_{ACT} when extrapolated to zero force. Plots of k_{ACT} versus force (Fig. S4) indicate that in our experiments, g_{APP} is ~2 s^{-1} independently of the phosphorylation level and the *ACTC* E361G mutation. Our t_{LIN} and k_{REL} measurements suggest that the time course of relaxation could be partly determined by the rate of Ca^{2+} dissociation in this system. Variations in the relative rates of cross-bridge detachment and Ca^{2+} dissociation in different experimental systems

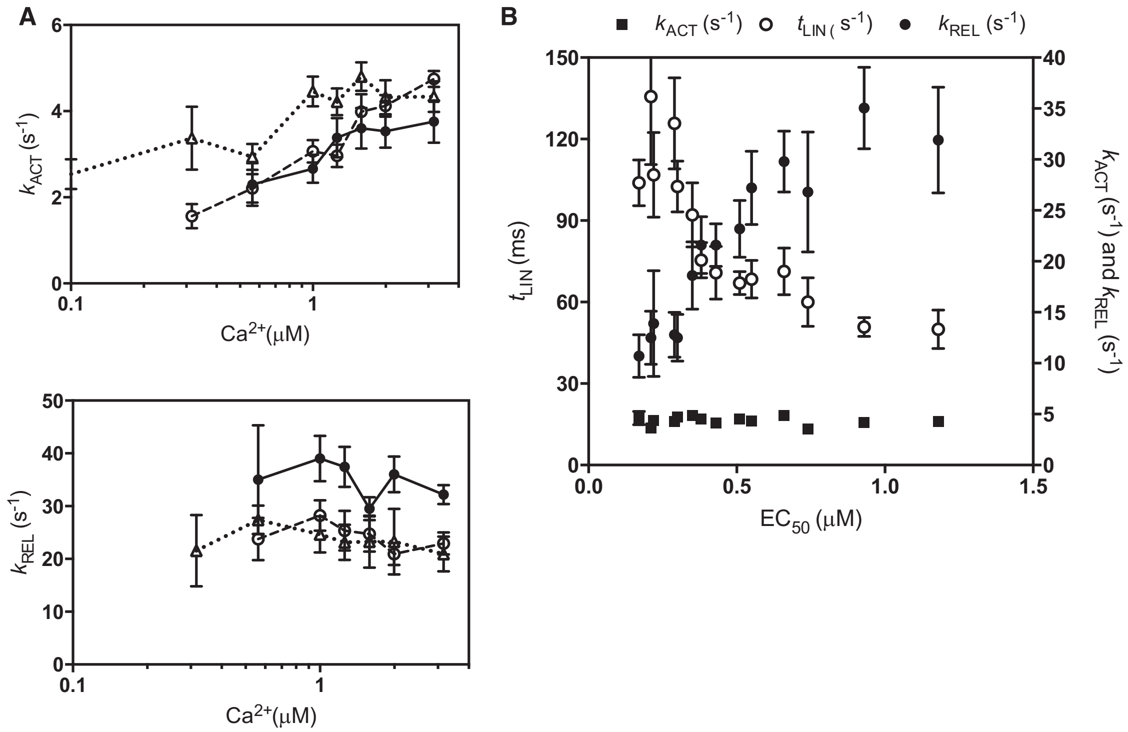

FIGURE 7 Ca^{2+} dependence of myofibril contraction and relaxation rates. (*A*) Relationship between Ca^{2+} and k_{ACT} and k_{REL} for phosphorylated NTG myofibrils (*solid circles*, *solid line*), unphosphorylated NTG myofibrils (*open circles, dashed line*), and phosphorylated *ACTC* E361G myofibrils (*open triangles, dotted line*) at SL = 2.17. Values are means ± SE. (*B*) Relationship among k_{ACT} (*solid squares*), t_{LIN} (*open circles*), k_{REL} (*solid circles*), and EC_{50} determined under 14 different conditions (data from Table 1).

could determine whether or not the relaxation rate is related to Ca^{2+} sensitivity.

Myofibrils are the simplest contractile system in which LDA can be measured. In our myofibrils, we observed LDA of similar magnitude to that reported in skinned myocytes and muscle fibers, thus confirming that LDA is sarcomere based (56–58). It is interesting to note that in our myofibrils, LDA was apparently independent of MyBP-C and troponin I phosphorylation and the DCM mutation *ACTC* E361G.

SUPPORTING MATERIAL

Five figures are available at http://www.biophysj.org/biophysj/supplemental/S0006-3495(14)01075-3.

We are grateful to Corrado Poggesi and Chiara Tesi (University of Florence) for help and advice on constructing the myofibrillar force apparatus.

This work was supported by grants from the British Heart Foundation (BHF RG/08/010/25918 and FS/09/024/24014).

REFERENCES

1. Gordon, A. M., E. Homsher, and M. Regnier. 2000. Regulation of contraction in striated muscle. *Physiol. Rev.* 80:853–924.
2. Solaro, R. J. 2002. Modulation of cardiac myofilament activity by protein phosphorylation. *In* Handbook of Physiology: Section 2: The Cardiovascular System. H. F. E. Page and R. J. Solaro, editors. Oxford University Press, New York, pp. 264–300.
3. Layland, J., R. J. Solaro, and A. M. Shah. 2005. Regulation of cardiac contractile function by troponin I phosphorylation. *Cardiovasc. Res.* 66:12–21.
4. England, P. J. 1976. Studies on the phosphorylation of the inhibitory subunit of troponin during modification of contraction in perfused rat heart. *Biochem. J.* 160:295–304.
5. Solaro, R. J., A. J. Moir, and S. V. Perry. 1976. Phosphorylation of troponin I and the inotropic effect of adrenaline in the perfused rabbit heart. *Nature.* 262:615–617.
6. al-Hillawi, E., D. G. Bhandari, …, I. P. Trayer. 1995. The effects of phosphorylation of cardiac troponin-I on its interactions with actin and cardiac troponin-C. *Eur. J. Biochem.* 228:962–970.
7. Mittmann, K., K. Jaquet, and L. M. Heilmeyer, Jr. 1990. A common motif of two adjacent phosphoserines in bovine, rabbit and human cardiac troponin I. *FEBS Lett.* 273:41–45.
8. Ayaz-Guner, S., J. Zhang, …, Y. Ge. 2009. In vivo phosphorylation site mapping in mouse cardiac troponin I by high resolution top-down electron capture dissociation mass spectrometry: Ser22/23 are the only sites basally phosphorylated. *Biochemistry.* 48:8161–8170.
9. Dong, W. J., J. J. Jayasundar, …, H. C. Cheung. 2007. Effects of PKA phosphorylation of cardiac troponin I and strong crossbridge on conformational transitions of the N-domain of cardiac troponin C in regulated thin filaments. *Biochemistry.* 46:9752–9761.
10. Zhang, R., J. Zhao, …, J. D. Potter. 1995. Cardiac troponin I phosphorylation increases the rate of cardiac muscle relaxation. *Circ. Res.* 76:1028–1035.
11. Kentish, J. C., D. T. McCloskey, …, R. J. Solaro. 2001. Phosphorylation of troponin I by protein kinase A accelerates relaxation and cross-bridge cycle kinetics in mouse ventricular muscle. *Circ. Res.* 88:1059–1065.

12. Fentzke, R. C., S. H. Buck, ..., J. M. Leiden. 1999. Impaired cardiomyocyte relaxation and diastolic function in transgenic mice expressing slow skeletal troponin I in the heart. *J. Physiol.* 517:143–157.

13. Yasuda, S., P. Coutu, ..., J. M. Metzger. 2007. Cardiac transgenic and gene transfer strategies converge to support an important role for troponin I in regulating relaxation in cardiac myocytes. *Circ. Res.* 101:377–386.

14. Pi, Y., K. R. Kemnitz, ..., J. W. Walker. 2002. Phosphorylation of troponin I controls cardiac twitch dynamics: evidence from phosphorylation site mutants expressed on a troponin I-null background in mice. *Circ. Res.* 90:649–656.

15. Marston, S. B. 2011. How do mutations in contractile proteins cause the primary familial cardiomyopathies? *J. Cardiovasc. Transl. Res.* 4:245–255.

16. Chang, A. N., and J. D. Potter. 2005. Sarcomeric protein mutations in dilated cardiomyopathy. *Heart Fail. Rev.* 10:225–235.

17. Morimoto, S. 2008. Sarcomeric proteins and inherited cardiomyopathies. *Cardiovasc. Res.* 77:659–666.

18. Memo, M., M.-C. Leung, ..., A. E. Messer. 2013. Familial dilated cardiomyopathy mutations uncouple troponin I phosphorylation from changes in myofibrillar Ca^{2+} sensitivity. *Cardiovasc. Res.* 99:65–73.

19. Song, W., E. Dyer, ..., S. B. Marston. 2011. Molecular mechanism of the E99K mutation in cardiac actin (ACTC gene) that causes apical hypertrophy in man and mouse. *J. Biol. Chem.* 286:27582–27593.

20. Marston, S. B., W. Song, ..., P. G. Vikhorev. 2013. DCM-causing mutation ACTC E361G blunts responses to adrenergic agonists, reduces cardiac reserve and predispose to heart failure under chronic stress in a transgenic mouse model. *J. Mol. Cell. Cardiol.* 65:s108.

21. Kooij, V., M. Saes, ..., G. J. Stienen. 2010. Effect of troponin I Ser23/24 phosphorylation on Ca^{2+}-sensitivity in human myocardium depends on the phosphorylation background. *J. Mol. Cell. Cardiol.* 48:954–963.

22. Messer, A. E., C. E. Gallon, ..., S. B. Marston. 2009. The use of phosphate-affinity SDS-PAGE to measure the cardiac troponin I phosphorylation site distribution in human heart muscle. *Proteomics Clin. Appl.* 3:1371–1382.

23. Walker, J. S., L. A. Walker, ..., P. de Tombe. 2011. Protein kinase A changes calcium sensitivity but not crossbridge kinetics in human cardiac myofibrils. *Am. J. Physiol. Heart Circ. Physiol.* 301:H138–H146.

24. Song, W., E. Dyer, ..., S. Marston. 2010. Investigation of a transgenic mouse model of familial dilated cardiomyopathy. *J. Mol. Cell. Cardiol.* 49:380–389.

25. Ayittey, P. N., J. S. Walker, ..., P. P. de Tombe. 2009. Glass microneedles for force measurements: a finite-element analysis model. *Pflugers Arch.* 457:1415–1422.

26. de Tombe, P. P., A. Belus, ..., C. Poggesi. 2007. Myofilament calcium sensitivity does not affect cross-bridge activation-relaxation kinetics. *Am. J. Physiol. Regul. Integr. Comp. Physiol.* 292:R1129–R1136.

27. Tesi, C., N. Piroddi, ..., C. Poggesi. 2002. Relaxation kinetics following sudden Ca(2+) reduction in single myofibrils from skeletal muscle. *Biophys. J.* 83:2142–2151.

28. Stehle, R., M. Krüger, and G. Pfitzer. 2002. Force kinetics and individual sarcomere dynamics in cardiac myofibrils after rapid Ca(2+) changes. *Biophys. J.* 83:2152–2161.

29. Song, W., P. G. Vikhorev, ..., N. A. Curtin. 2013. Mechanical and energetic properties of papillary muscle from ACTC E99K transgenic mouse models of hypertrophic cardiomyopathy. *Am. J. Physiol. Heart Circ. Physiol.* 304:H1513–H1524.

30. Wang, Y., J. R. Pinto, ..., J. D. Potter. 2012. Generation and functional characterization of knock-in mice harboring the cardiac troponin I-R21C mutation associated with hypertrophic cardiomyopathy. *J. Biol. Chem.* 287:2156–2167.

31. Marston, S. B., and P. P. de Tombe. 2008. Troponin phosphorylation and myofilament Ca^{2+}-sensitivity in heart failure: increased or decreased? *J. Mol. Cell. Cardiol.* 45:603–607.

32. Jweied, E., P. deTombe, and P. M. Buttrick. 2007. The use of human cardiac tissue in biophysical research: the risks of translation. *J. Mol. Cell. Cardiol.* 42:722–726.

33. Biesiadecki, B. J., T. Kobayashi, ..., P. P. de Tombe. 2007. The troponin C G159D mutation blunts myofilament desensitization induced by troponin I Ser23/24 phosphorylation. *Circ. Res.* 100:1486–1493.

34. Huke, S., and B. C. Knollmann. 2010. Increased myofilament Ca^{2+}-sensitivity and arrhythmia susceptibility. *J. Mol. Cell. Cardiol.* 48:824–833.

35. Stelzer, J. E., J. R. Patel, and R. L. Moss. 2006. Protein kinase A-mediated acceleration of the stretch activation response in murine skinned myocardium is eliminated by ablation of cMyBP-C. *Circ. Res.* 99:884–890.

36. Rao, V. S., F. S. Korte, ..., D. A. Martyn. 2013. N-terminal phosphorylation of cardiac troponin-I reduces length-dependent calcium sensitivity of contraction in cardiac muscle. *J. Physiol.* 591:475–490.

37. Inoue, T., F. Kobirumaki-Shimozawa, ..., N. Fukuda. 2013. Depressed Frank-Starling mechanism in the left ventricular muscle of the knock-in mouse model of dilated cardiomyopathy with troponin T deletion mutation ΔK210. *J. Mol. Cell. Cardiol.* 63:69–78.

38. Howarth, J. W., J. Meller, ..., P. R. Rosevear. 2007. Phosphorylation-dependent conformational transition of the cardiac specific N-extension of troponin I in cardiac troponin. *J. Mol. Biol.* 373:706–722.

39. Baryshnikova, O. K., I. M. Robertson, ..., B. D. Sykes. 2008. The dilated cardiomyopathy G159D mutation in cardiac troponin C weakens the anchoring interaction with troponin I. *Biochemistry.* 47:10950–10960.

40. Solaro, R. J., P. Rosevear, and T. Kobayashi. 2008. The unique functions of cardiac troponin I in the control of cardiac muscle contraction and relaxation. *Biochem. Biophys. Res. Commun.* 369:82–87.

41. Bayliss, C. R., A. M. Jacques, ..., A. E. Messer. 2013. Myofibrillar Ca(2+) sensitivity is uncoupled from troponin I phosphorylation in hypertrophic obstructive cardiomyopathy due to abnormal troponin T. *Cardiovasc. Res.* 97:500–508.

42. Takeda, S., A. Yamashita, ..., Y. Maéda. 2003. Structure of the core domain of human cardiac troponin in the Ca(2+)-saturated form. *Nature.* 424:35–41.

43. Vinogradova, M. V., D. B. Stone, ..., R. J. Fletterick. 2005. Ca(2+)-regulated structural changes in troponin. *Proc. Natl. Acad. Sci. USA.* 102:5038–5043.

44. Dyer, E. C., A. M. Jacques, ..., S. B. Marston. 2009. Functional analysis of a unique troponin c mutation, GLY159ASP, that causes familial dilated cardiomyopathy, studied in explanted heart muscle. *Circ Heart Fail.* 2:456–464.

45. Robinson, P., P. J. Griffiths, ..., C. S. Redwood. 2007. Dilated and hypertrophic cardiomyopathy mutations in troponin and alpha-tropomyosin have opposing effects on the calcium affinity of cardiac thin filaments. *Circ. Res.* 101:1266–1273.

46. Gramlich, M., B. Michely, ..., B. Gerull. 2009. Stress-induced dilated cardiomyopathy in a knock-in mouse model mimicking human titin-based disease. *J. Mol. Cell. Cardiol.* 47:352–358.

47. Piroddi, N., A. Belus, ..., C. Poggesi. 2007. Tension generation and relaxation in single myofibrils from human atrial and ventricular myocardium. *Pflugers Arch.* 454:63–73.

48. Lopez-Davila, A. J., F. Elhamine, ..., R. Stehle. 2012. Kinetic mechanism of Ca^{2+}-controlled changes of skeletal troponin I in psoas myofibrils. *Biophys. J.* 103:1254–1264.

49. Stehle, R., J. Solzin, ..., C. Poggesi. 2009. Insights into the kinetics of Ca^{2+}-regulated contraction and relaxation from myofibril studies. *Pflugers Arch.* 458:337–357.

50. Iorga, B., N. Blaudeck, ..., R. Stehle. 2008. Lys184 deletion in troponin I impairs relaxation kinetics and induces hypercontractility in murine cardiac myofibrils. *Cardiovasc. Res.* 77:676–686.

51. Robertson, S. P., J. D. Johnson, …, R. J. Solaro. 1982. The effect of troponin I phosphorylation on the Ca^{2+}-binding properties of the Ca^{2+}-regulatory site of bovine cardiac troponin. *J. Biol. Chem.* 257:260–263.

52. Dong, W. J., J. Xing, …, H. C. Cheung. 2008. Structural kinetics of cardiac troponin C mutants linked to familial hypertrophic and dilated cardiomyopathy in troponin complexes. *J. Biol. Chem.* 283:3424–3432.

53. Poggesi, C., C. Tesi, and R. Stehle. 2005. Sarcomeric determinants of striated muscle relaxation kinetics. *Pflugers Arch.* 449:505–517.

54. Lehrer, S. S., and M. A. Geeves. 1998. The muscle thin filament as a classical cooperative/allosteric regulatory system. *J. Mol. Biol.* 277: 1081–1089.

55. Little, S. C., B. J. Biesiadecki, …, J. P. Davis. 2012. The rates of Ca^{2+} dissociation and cross-bridge detachment from ventricular myofibrils as reported by a fluorescent cardiac troponin C. *J. Biol. Chem.* 287:27930–27940.

56. Konhilas, J. P., T. C. Irving, and P. P. de Tombe. 2002. Length-dependent activation in three striated muscle types of the rat. *J. Physiol.* 544:225–236.

57. de Tombe, P. P., R. D. Mateja, …, T. C. Irving. 2010. Myofilament length dependent activation. *J. Mol. Cell. Cardiol.* 48:851–858.

58. McDonald, K. S., L. J. Field, …, R. L. Moss. 1995. Length dependence of Ca^{2+} sensitivity of tension in mouse cardiac myocytes expressing skeletal troponin C. *J. Physiol.* 483:131–139.

SUPPORTING MATERIAL

2014BIOPHYSJ304024:

"The DCM-causing mutation ACTC E361G in cardiac muscle myofibrils specifically abolishes modulation of Ca2+-regulation by phosphorylation of troponin I"

Petr G. Vikhorev, Weihua Song, Ross Wilkinson, O'Neal Copeland, Andrew E. Messer, Michael A. Ferenczi, Steven B. Marston

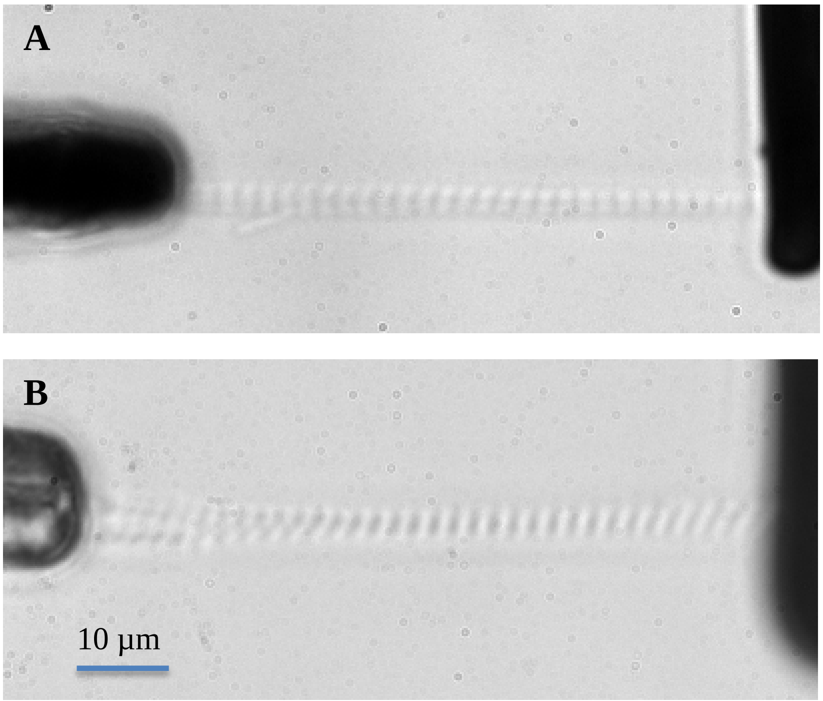

FIGURE S1

A small bundle of myofibrils positioned horizontally between two microneedles

A small group of myofibrils (3-4 myofibrils; *A*, wild-type; *B*, *ACTC* E361G) arranged in a stack were pre-stretched to a SL of 2.17 µm. Due to force probe compliance, the average SL of contracted myofibrils at maximum force became 2.07 and 1.82 µm, respectively for the myofibrils pre-stretch to a SL of 2.17 and 1.90. The changes in SL were about 2-6% in all conditions.

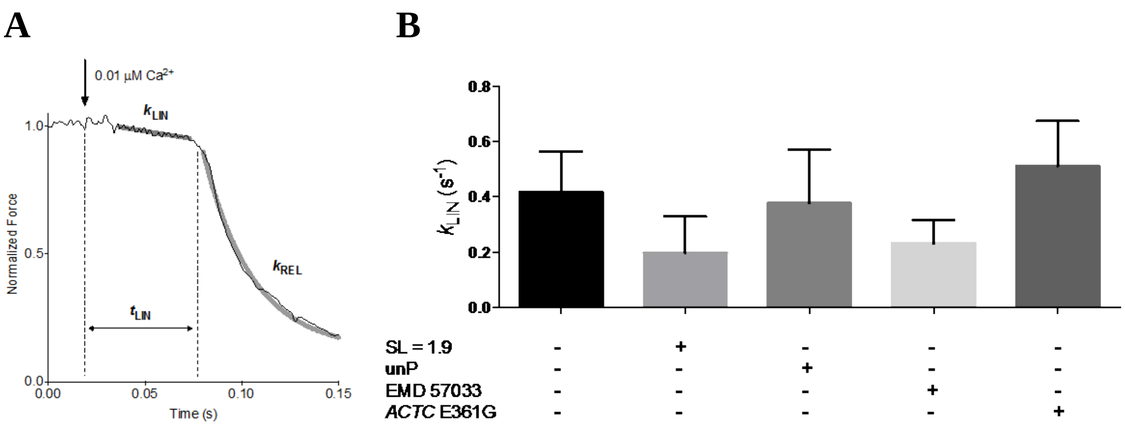

FIGURE S2

The effect of SL change, dephosphorylation, EMD 57033 and mutation *ACTC* E361G on the parameter k_{LIN}.

(*A*) During the slow relaxation phase an active tension is essentially not changing. After a certain time lag (t_{LIN}) the slow phase (characterized also by a rate constant k_{LIN}) is followed by a rapid exponential force decay with a rate constant k_{REL}. A short transition period can be seen during which the system shifts from the linear phase to the exponential. The transition phase was not entered neither the regression line calculation nor the exponential. The k_{LIN} was calculated from the slope of the linear fit (k_{LIN}=slope/Fmax).

(*B*) The k_{LIN} measured in different conditions, compared to control (first black column in *B*; NTG myofibrils, SL = 2.17, phosphorylated, untreated). Values are means ± SE, n = 5-9. There was no difference ($p \geq 0.35$) in k_{LIN}.

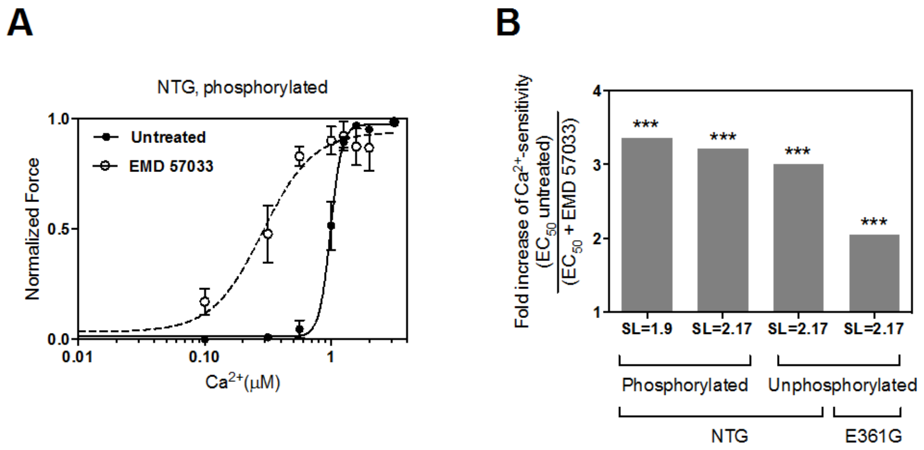

FIGURE S3

The effect of EMD57033 on mouse myofibril contractility

(*A*) The effect of EMD57033 on force-Ca^{2+} relationship. The Ca^{2+}-force sensitivity curve for treated myofibrils (*open circles*, *dashed line*) is shifted to the left, as compared to untreated NTG myofibrils (*solid circles*, *solid line*). Sarcomere length was 2.17 µm. (*B*) The increase in Ca^{2+} sensitivity ($1/EC_{50}$) resulting from incubation with EMD57033. Values are means. ***P < 0.001. Data from Table 1.

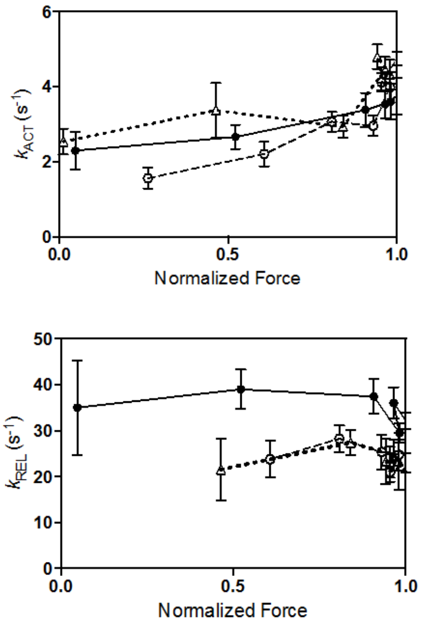

FIGURE S4

Relationship of myofibril contraction and relaxation rates to developed force

Relationship between Force and k_{ACT} and k_{REL} for phosphorylated NTG myofibrils (*solid circles*, *solid line*), unphosphorylated NTG myofibrils (*open circles*, *dashed line*) and phosphorylated *ACTC* E361G myofibrils (*open triangles*, *dotted line*) at SL=2.17. Values are means ± SE.

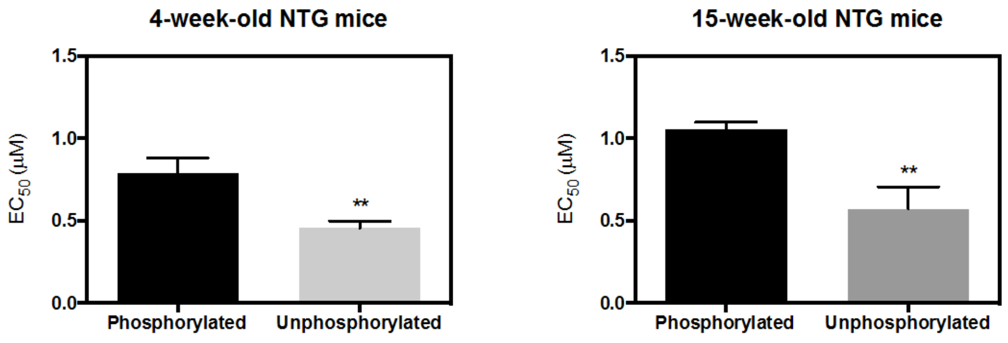

FIGURE S5

The effect of mouse age on myofibril Ca^{2+}-sensitivity

Comparison of EC_{50} values between phosphorylated and unphosphorylated myofibrils from 4-week-old (*A*) and 28-week-old (*B*). Values are means ± SE, n = 5-8. **P < 0.01.

Phosphorylated myofibrils from 4-week-old mouse had slightly higher calcium sensitivity of force than myofibrils obtained from 28-week-old mouse (0.79 ± 0.09 vs. 1.05 ± 0.04 µM, p = 0.02). There was no difference in *n*H. Dephosphorylation of sarcomere proteins increased calcium sensitivity by 1.73 and 1.84 fold in 4-week-old and 28-week-old mice, respectively. The age difference between unphosphorylated samples was not significant (p=0.37).
